# African American Children’s Diminished Returns of Subjective Family Socioeconomic Status on Fun Seeking

**DOI:** 10.3390/children7070075

**Published:** 2020-07-09

**Authors:** Shervin Assari, Golnoush Akhlaghipour, Shanika Boyce, Mohsen Bazargan, Cleopatra H. Caldwell

**Affiliations:** 1 Department of Family Medicine, Charles R Drew University of Medicine and Science, Los Angeles, CA 90059, USA; Mohsenbazargan@cdrewu.edu; 2 Department of Neurology, University of California Los Angeles, Los Angeles, CA 90095, USA; golnoush.akhlaghi@gmail.com; 3 Department of Pediatrics, Charles R Drew University of Medicine and Science, Los Angeles, CA 90059, USA; ShanikaBoyce@cdrewu.edu; 4 Center for Research on Ethnicity, Culture, and Health (CRECH), School of Public Health, University of Michigan, Ann Arbor, MI 48104, USA; cleoc@umich.edu; 5 Department of Health Behavior and Health Education, School of Public Health, University of Michigan, Ann Arbor, MI 48104, USA

**Keywords:** education, parenting, socioeconomic status, children, adolescents, risk behaviors, emotion regulation

## Abstract

*Background:* Reward sensitivity (fun-seeking) is a risk factor for a wide range of high-risk behaviors. While high socioeconomic status (SES) is known to reduce reward sensitivity and associated high-risk behaviors, less is known about the differential effects of SES on reward sensitivity. It is plausible to expect weaker protective effects of family SES on reward sensitivity in racial minorities, a pattern called Minorities’ Diminished Returns (MDRs). *Aim:* We compared Caucasian and African American (AA) children for the effects of subjective family SES on children’s fun-seeking. *Methods:* This was a cross-sectional analysis of 7061 children from the Adolescent Brain Cognitive Development (ABCD) study. The independent variable was subjective family SES. The main outcome was children’s fun-seeking measured by the behavioral approach system (BAS) and behavioral avoidance system (BIS). Age, gender, marital status, and household size were the covariates. *Results:* In the overall sample, high subjective family SES was associated with lower levels of fun-seeking. We also found a statistically significant interaction between race and subjective family SES on children’s fun-seeking in the overall sample, suggesting that high subjective family SES is associated with a weaker effect on reducing fun-seeking among AA than Caucasian children. In race-stratified models, high subjective family SES was protective against fun-seeking of Caucasian but not AA children. *Conclusion:* Subjective family SES reduces the fun-seeking for Caucasian but not AA children.

## 1. Introduction

In their earlier [1] and later [2] theoretical work on the behavioral approach system (BAS) and reinforcement sensitivity theory (RST), Gray and McNaughton introduced and discussed the contribution of the BAS-based sensitivity to reward as the main driver of human behaviors [3]. These BAS-based traits correlate with traits such as impulsivity and risk taking [4]. The BAS-based sensitivity to reward is also shown to predict a wide range of high-risk behaviors such as tobacco use [5,6,7,8,9,10], alcohol use [11,12,13,14], emotional eating [15], obesity [16], aggression [17], and sexual risk [18,19]. These traits are also linked to several psychiatric disorders, such as bipolar disorder, anxiety, depression, and post-traumatic stress disorder (PTSD) [20]. Some evidence suggests that similar to high-risk behaviors [21] and impulsivity [22,23], BAS-traits may also be linked to race and socioeconomic status (SES), with African Americans (AAs) and individuals with lower SES scoring higher in reward-seeking traits compared to Caucasians and high SES people [24].

Based on Gray’s reinforcement sensitivity theory (RST) [1], certain traits that reflect neurobiological sensitivity to rewards guide human emotion, motivation, and behavior. Rooted in the BAS developed by Carver and White [16], high reward sensitivity reflects individuals’ high sensitivity to conditioned cues, which signal the individual about a higher-than-luck probability of reward. Individuals with a high score on the trait reward sensitivity are more likely to act on any cues that are linked to an internal or external reward. In a more recent version of the theory [2], Gray and McNaughton discussed the contribution of the BAS-based reward sensitivity and approach-related behaviors and stimuli to human behaviors. Many investigators have found evidence regarding the relevance of reward sensitivity, as the main component of approach motivations, to a wide range of health and behavioral outcomes in clinical samples [24,25,26], as well as the general population [27]. These BAS-based reward-sensitivity traits are also relevant to children’s risk behaviors [11,15,28]. Fun-seeking is one of the least-studied aspects of BAS-reward-sensitivity domains.

Research has shown that AA children are at an increased risk of high-risk behaviors that can be linked to reward-dependency, relative to their Caucasian counterparts. For example, in comparison to Caucasian children, AA children report more risk in the domains of aggression [29], sexual debut [30], and academic and school performance [31]. As these risky behaviors operate as barriers against positive health and economic outcomes later in life [32,33,34,35]; it is essential to study environmental and psychological factors that explain high reward sensitivity (and associated risky behaviors) of AA children. Such knowledge may inform public and social policies that may leverage interventions during adolescence to eliminate later racial inequalities [32,33,34,35].

Given the close overlap between race and parental education in the US [36], researchers have shown immense interest in understanding how race and parental education have combined effects on children and adults’ health inequalities [37,38,39]. As both racial minority status and low SES reflect food and housing insecurity, economic adversities, stress, and financial difficulties [40,41,42,43], some of the effects of race and parental education overlap. Thus, parental education carries some of the effects of race on children’s outcomes [36]. However, more recent data show that the effect of race and parental education is more complex, as they show mediational and moderation effects on health inequalities [44,45,46,47]. While parental education should be a proxy of access to buffers and protective factors [44,45,46,47], these protective effects of parental education seem to be weaker for AA than Caucasian children.

There are at least two complementary theories that provide an explanation of how race and parental education jointly impact children’s high risk, cognitive, and health outcomes. The first theory, dominant in the literature, and used commonly as a traditional explanation of the inequalities, attributes the observed racial gap in children’s outcomes to the racial differences in parental education and other family SES indicators [36,48,49,50]. In a statistical term, this theory conceptualizes parental education as a mediator of (why) race influences children’s outcomes [51,52,53]. If this theory is followed, then the researcher would recommend that enhancing family SES and closing the racial gap in parental education should be taken as the main strategy for closing the racial gap in children’s health. Some example solutions are income redistribution policies, tax policies, and helping racial minorities to accumulate wealth [54,55].

Minorities’ Diminished Returns (MDRs) [56,57], the second theory, however, argues that the effects of parental education and other family SES indicators are always weaker for any racial minority group, such as AAs, compared to Caucasians. This line of view is not against the traditional mediational model, but provides an explanation for why despite years of investment, and despite a decline in the gap between races in terms of parental education, the racial gap in health is still sustained, and in some cases, widened. The MDRs theory has been supported by a growing body of evidence showing that parental education [58], family income [59,60], and marital status [61] generate less health and well-being for AA than Caucasian children. This literature is repeatedly shown for emotional and behavioral outcomes [58,59,60,62,63]. For example, high family SES showed a smaller effect as a preventive factor on impulsivity [59], depression [62], anxiety [64], aggression [58], grade point average (GPA) [58,65,66], and substance use [58] for AA than Caucasian children. Similarly, high SES AA youth are found to be at high risk of ADHD [67] and obesity [68]. Given the existing MDRs, it would be too optimistic and unrealistic to expect racial inequities to disappear if we could fully eliminate SES inequalities. In this view, SES indicators such as parental education are seen as both remedies and also a source of inequalities across racial groups. While it is essential to eliminate the SES gap, we should complement our policy responses in a specific way that particularly empowers AA families to mobilize their resources and secure health outcomes [56,57].

As described above, the MDR literature suggests that educational attainment of one’s own [69] and one’s parents [70,71,72] generate fewer tangible outcomes for racial minorities such as AAs. This might be because AAs and Caucasians differ in getting chances and opportunities to mobilize their education and secure high paying jobs in the presence of high education [57,59,64,71,73,74]. As a result of these MDRs of parental education, compared to their Caucasian counterparts, AA children with highly educated parents show worse than expected outcomes that are disproportionate to their family SES [56,57,59,60,63].

To extend the existing knowledge on the contribution of reward sensitivity as a mechanism that may explain some of the MDRs, we compared AA and Caucasian children for the effect of subjective family SES, a strong family SES determinant of children’s risk behaviors and health, with fun-seeking, a trait that may be linked to high-risk behaviors such as aggression, tobacco use, alcohol use, sexual initiation, and impulsivity. In this study, we explored the combined effects of race and subjective family SES on children’s reward sensitivity (fun-seeking). We expected a weaker effect of high subjective family SES on reducing reward sensitivity (i.e., fun-seeking) for AA than Caucasian children.

## 2. Methods

### 2.1. Design and Settings

We performed a secondary analysis of data from the Adolescent Brain Cognitive Development (ABCD) study [75,76,77,78,79], a landmark children and adolescents brain development study in the United States. Detailed information on the details of the ABCD study is available elsewhere [75,80].

### 2.2. Participants and Sampling

Participants of the ABCD study were children of age 9–10 years. Children in the ABCD study were recruited from multiple cities across the states. Overall, there were 21 sites that recruited children to the ABCD study. The recruitment of the ABCD sample was mainly done through school systems. A detailed description of the ABCD sampling is available here [81]. Four thousand one hundred eighty-eight participants entered our analysis. Eligibility for our analysis had valid data on race, parental education, marital status, reward sensitivity (fun-seeking), and being AA or Caucasian. The analytical sample of this paper was 7061.

### 2.3. Study Variables

The study variables included race, demographic factors, subjective family SES, parental marital status, and reward sensitivity (fun-seeking).

#### 2.3.1. Outcome

Fun-Seeking. In this study, fun-seeking was measured using the behavioral approach system (BAS) [3], which was based on the Carver model of the reinforcement sensitivity theory (RST) [16]. The defined reward-seeking is a trait closely linked to impulsivity and risk taking [4], with considerable implications for a wide range of high-risk behaviors such as tobacco use [5,6,7,8,9,10], alcohol use [11,12,13,14], emotional eating [15], obesity [16], aggression [17], and sexual risk [18,19]. Based on Gray’s reinforcement sensitivity theory (RST) [1], a high score on the reward sensitivity trait reflects individuals’ high sensitivity to conditioned cues, which signal the individual about a higher-than-luck probability of reward. We operationalized BAS-based reward sensitivity (fun-seeking), which was a continuous measure.

#### 2.3.2. Moderator

Race. Race was self-identified. Race was a categorical variable and coded 1 for AAs and 0 for Caucasians (reference category).

#### 2.3.3. Independent Variable

Subjective Family SES. This study measured subjective family SES using the following seven items. Participants were asked “In the past 12 months, has there been a time when you and your immediate family experienced any of the following:” (1) “Needed food but couldn’t afford to buy it or couldn’t afford to go out to get it?”, (2) “Were without telephone service because you could not afford it?”, (3) “Didn’t pay the full amount of the rent or mortgage because you could not afford it?”, (4) “Were evicted from your home for not paying the rent or mortgage?”, (5) “Had services turned off by the gas or electric company, or the oil company wouldn’t deliver oil because payments were not made?”, (6) “Had someone who needed to see a doctor or go to the hospital but didn’t go because you could not afford it?”, and (7) “Had someone who needed a dentist but couldn’t go because you could not afford it?” Responses were 0 or 1. We calculated a mean score (a continuous measure), which ranged between 0 and 1 with a higher score indicating higher subjective family SES. Subjective family SES is an accepted SES indicator, as it reflects some aspects of the SES that are not captured by objective SES indicators [82,83,84,85,86,87,88]. Subjective SES is shown to have some health effects that are not seen by objective SES [82,84,85,89,90,91].

#### 2.3.4. Confounders

Age, sex, household size, and parental marital status were the confounders. Parents reported the age of their children. Age (years) was calculated as the difference between the date of birth to the date of the enrolment to the study. Sex was a dichotomous variable: males = 1, females = 0. Parental marital status was a dichotomous variable. This variable was self-reported by the parent who was interviewed. This variable was coded as married = 1 vs. other = 0. Household size, reported by the parent, was a continuous measure.

### 2.4. Data Analysis

We used the statistical package SPSS to perform our data analysis. Mean (standard deviation [SD]) and frequency (%) were described depending on the variable type. We also performed the Chi-square and independent-sample t-test for our bivariate analysis. For our multivariable modeling, we performed four linear regression models. Our first two models were performed in the overall sample. Model 1 was performed without the interaction terms. Model 2 added an interaction term between race and subjective family SES. Then we performed two additional models specifically for race (race-stratified models). Model 3 was tested in Caucasian, and Model 4 was tested in AA children. Our models used age, sex, household size, and marital status as the covariates. Unstandardized regression coefficient (b), SE, 95% CI, t value, and *p*-value were reported for each model. *p* values equal to or less than 0.05 were statistically significant.

### 2.5. Ethical Aspect

The ABCD study received an Institutional Review Board (IRB) approval from the University of California, San Diego (UCSD). Each child provided assent. Each parent signed an informed consent [80]. As this analysis was performed on fully de-identified data, the study was found to be non-human subject research. Thus, our analysis was exempt from a full IRB review.

## 3. Results

### 3.1. Descriptives

Overall, 7061, 8–11-year-old children entered into this analysis. From this number, most were Caucasians (*n* = 5097; 72.2%) and the rest were AAs (*n* = 1964; 27.8%). Table 1 presents a summary of the descriptive statistics for the pooled sample. AAs had lower subjective family SES than Caucasians.

### 3.2. Multivariate Analysis (Pooled Sample)

Table 2 shows the results of two linear regression models in the overall (pooled) sample. Model 1 (Main Effect Model) showed a protective effect of high subjective family SES against reward sensitivity (fun-seeking). Model 2 (Interaction Model) showed a statistically significant interaction between race and subjective family SES on reward sensitivity (fun-seeking), which was suggestive of a weaker protective effect of high subjective family SES on reward sensitivity (fun-seeking) for AA children relative to Caucasian children. The only factor that was not significant was household size.

### 3.3. Multivariate Analysis (Race-Stratified Models)

Table 3 shows the results of two linear regression models in racial groups. Model 3 (Caucasians) showed protective effects of high subjective family SES on reward sensitivity (fun-seeking) of Caucasian children. Model 4 (AAs) did not show any protective effect of high subjective family SES on reward sensitivity (fun-seeking) for AA children.

## 4. Discussion

In this study, while high subjective family SES was associated with lower reward sensitivity (fun-seeking) overall, this was only true for Caucasian but not AA children. Similar results were observed for the models in the pooled sample as those stratified by race. Thus, racial minority status (AA) bounds the boosting effect of subjective family SES on reducing the reward sensitivity (fun-seeking) for American children. As a result, AA children report high reward sensitivity (fun-seeking) across all SES levels.

The observed diminished returns of subjective family SES on reward sensitivity (fun-seeking) is very similar to the previous publications on the MDRs of parental education and income on impulsivity [59], ADHD [67], inhibitory control [92], social problems [93], emotional problems [93], behavioral problems [93], anxiety [64], depression [62], aggression [58], GPA [58,65,66], and substance use [58]. These are all examples of diminishing returns of family SES for AA compared to Caucasian youth [69,73,94,95].

MDRs are due to society and not due to the culture, behavior, or weakness of the marginalized group. These patterns are not specific to AAs [60], and can be seen for all marginalizing identities [56,57] such as Hispanics [69,96,97,98], Asian Americans [99], Native Americans [100], LGBTQs [94], immigrants [101], or even marginalized Caucasians [102]. They are also not specific to an age group, as similar results are shown for children [59,60,63], adults [73], and older adults [103]. Finally, these MDRs are relevant to all SES indicators such as subjective SES [85], education [69], income [59], employment [104], marital status [64], and even non-economic coping skills such as self-efficacy [105,106].

Various sociological, economic, and behavioral mechanisms are involved in explaining the MDRs of family SES on reward sensitivity for AA families. AA families experience high levels of economic, social, general, and race-related stress across all SES levels [107]. Upward social mobility is differently taxing for AA and Caucasian families [108]. As SES increases, exposure [109,110,111,112,113] and vulnerability [85] to discrimination increases for AA families. This is in part because, for AA families, an increase in SES means higher closeness to Caucasians, which increases their exposure to discrimination [109,110]. As shown by a robust literature, discrimination is a risk factor for poor brain development, high-risk behaviors, and poor health [85,112,114]. As such, high SES AAs will show worse than expected outcomes across the board.

Residential segregation results in differences in the context in which AA and Caucasian families live, play, and worship. Due to segregation, school options are qualitatively different for AA and Caucasian families who may have identical SES. While Caucasian families live in high SES suburban neighborhoods and send their children to high-quality schools, African Americans are likely to live in a worse neighborhood, across all SES levels. Children of high SES AA families attend worse schools with lower resources [65,66,115]. That means the very same SES indicator will have differential effects on education and schooling for Caucasian and AA families. This means that high SES differently impacts school options and choices of Caucasian and AA families [116].

While lower SES of AAs is one type of disadvantage, MDRs reflect another class of disadvantage [56,57]. While the first one is about low family SES, MDRs are reflective of unequal outcomes across SES levels. Thus, researchers and policymakers should not only address inequality in SES, but they should also address inequality in the returns of SES. AAs are at a double disadvantage because they are affected by low SES and low return of the existing SES resources [56,117].

Multilevel economic, psychological, and societal mechanisms may be involved in explaining racial gaps in the returns of parental education [56,117]. MDRs may be due to racism across multiple societal institutions and social structures [56,117]. Racial prejudice interferes with the processes that are needed to gain benefits from available SES resources [118,119,120]. MDRs of educational attainment may be in part due to a history of childhood poverty [121]. The current study, however, did not explore societal and contextual processes that could explain such MDRs.

AA families are more likely to stay in poor neighborhoods despite high SES. Highly educated AAs are more likely to stay poor than Caucasians [72,122]. Similarly, AA families from high SES backgrounds may remain at a higher risk of environmental exposures than Caucasians with similar SES [109,110,112,123,124,125,126,127]. Similarly, high SES AA children spend time with peers with higher risk and behavioral problems than Caucasians with the same SES [58,99].

As this study shows, health disparities are not all due to SES differences, and some can be seen across the whole SES spectrum. This means that it is not race or SES but race and SES that shape health disparities [128,129,130]. The implication of these MDRs is that merely equalizing access to SES is not enough to tackle racial inequalities. Not only should SES gaps be eliminated or reduced, but there is a need to increase the degree to which SES can result in outcomes for AA families. To do so, policymakers should think about societal, social, environmental, and structural barriers that generate MDRs by reducing African Americans’ ability to leverage their resources. A real solution to MDRs-related disparities should be different from a solution of those that are caused by the SES-gaps. While the policy solution to health disparities due to SES gap is to increase AAs’ access to SES resources, a true and sustainable remedy to the MDRs-related inequalities is to reduce structural barriers so AA families can efficiently and effectively translate their SES and human capital and secure tangible outcomes. This is not possible unless we equalize the daily living conditions of AAs and Caucasians.

By reading the title, some readers may think we are blaming the African community for their behavior or fun-seeking behavior. That view would be racist and ignoring the fact that African Americans’ situation is the result of centuries of oppression and inequalities following slavery and Jim Crow. We also did not have the presumption that “fun-seeking” is a fundamental part of the culture of a specific racial group. In fact, we see culture as shaped by social environment. We think under the existing racism, reducing behaviors of a group to cultural differences would be still racist and unfair to the disadvantaged group. Still, a construct such as fun-seeking may be more relevant to one culture than another. We do not interpret this as one group is more impulsive or attracted by mundane aspects of life or pleasure. We simply built this study based on the MDRs and showed that, possibly due to racism, the slope of the effect of parental education on fun-seeking is lower in African American than Caucasian communities. We also do not believe any vertical evaluation of the two racial groups. In this view, we distance ourselves from any attribution of the findings to implying that Caucasians are the norm, or African Americans as different or less human. Thus, this paper should not be seen as a paper that generates divides between races (White superiority, White fragility, or any related beliefs). Our ultimate goal is to understand social factors and policies that can bring racial equality.

## 5. Limitations

This study, like any other study, comes with a specific set of methodological limitations. Cross-sectional studies such as ours are not appropriate for drawing causal inferences. Thus, our findings are indicative of the association between race, family SES, and children’s reward sensitivity (fun-seeking). Similarly, we only tested the MDRs of subjective family SES. Future research should test if MDRs go beyond subjective SES and also hold for parental education, income, wealth, class, occupational prestige, and other objective SES measures. In addition, this study only included individual-level SES indicators. It is important to test if the same rules also apply to neighborhood SES indicators such as home values and median income. Finally, this study only described the MDRs of family SES on reward sensitivity (fun-seeking) and did not seek the contextual factors that cause such MDRs. Also, it did not test if these MDRs mediate MDRs of risk-taking.

## 6. Conclusions

Relative to their Caucasian counterparts, AA children show lower SES and higher reward sensitivity (fun-seeking), which both operate as risk factors for a wide range of outcomes. These adversities in AA youth are compound by another profound and systemic disadvantage, which is a weaker effect of family SES on children’s reward sensitivity (fun-seeking). It is a result of the latter disadvantage that AA children show high reward sensitivity (fun-seeking) across all parental education levels. Thus, AA children show high-risk behaviors across all SES levels. In other terms, racial inequalities in reward sensitivity (fun-seeking) show a spill-over effect on middle and upper-class AAs. To minimize the racial gap in brain development and risk-taking behaviors that impact AAs, we need to address societal barriers that diminish the returns of family SES resources in securing outcomes for AA families. We need public and social policies that target structural and societal barriers, such as the unequal distribution of opportunities and resources.

## Figures and Tables

**Table 1:** Descriptive data overall and by race.

	All (*n* = 7061)	Caucasian (*n* = 5097)	African American (*n* = 1964)
	*n* (%)	*n* (%)	*n* (%)
**Sex**			
Male	3413 (48.3)	2430 (47.7)	983 (50.1)
Female	3648 (51.7)	2667 (52.3)	981 (49.9)
**Household Marital status** *			
Unmarried Household	2246 (31.8)	906 (17.8)	1340 (68.2)
Married Household	4815 (68.2)	4191 (82.2)	624 (31.8)
	**Mean (SD)**	**Mean (SD)**	**Mean (SD)**
Age (Year)	9.47(0.51)	9.47 (0.50)	9.47 (0.52)
Household Size	4.70(1.52)	4.72 (1.40)	4.63 (1.80)
Subjective Family SES *	0.93(0.16)	0.96 (0.12)	0.85 (0.22)
Fun-Seeking (Reward Sensitivity) *	5.67(2.67)	5.50 (2.59)	6.12 (2.81)

SES = Socioeconomic Status, SD = Standard Deviation, * *p* < 0.05 for comparison of African American and Caucasian children.

**Table 2:** Summary of linear regressions overall (*n* = 7061).

	Model 1 Main Effects						Model 2 Interaction Effects					
	b	SE	95% CI		t	*p*	b	SE	95% CI		t	*p*
Race (African Americans)	0.46	0.08	0.29	0.62	5.50	0.000	−0.75	0.38	−1.50	0.00	−1.96	0.050
Sex (Male)	0.40	0.06	0.28	0.53	6.38	0.000	0.40	0.06	0.28	0.53	6.36	0.000
Age	−0.19	0.06	−0.31	−0.07	−3.09	0.002	−0.19	0.06	−0.31	−0.07	−3.09	0.002
Household Size	−0.01	0.02	−0.05	0.04	−0.33	0.741	−0.01	0.02	−0.05	0.03	−0.40	0.689
Married household	−0.26	0.08	−0.43	−0.10	−3.18	0.001	−0.25	0.08	−0.41	−0.08	−2.96	0.003
Subjective Family SES	−0.45	0.21	−0.86	−0.03	−2.12	0.034	−1.17	0.31	−1.78	−0.57	−3.82	0.000
Subjective Family SES × Race							1.33	0.41	0.53	2.14	3.24	0.001
Constant	7.79	0.63	6.55	9.03	12.31	0.000	8.48	0.67	7.17	9.79	12.70	0.000

b = Unstandardized Regression Coefficient, SES = Socioeconomic Status, SE = Standard Error, CI = Confidence Interval.

**Table 3:** Summary of linear regressions by race (*n* = 7061).

	Model 1 Caucasians						Model 2 African Americans					
	**b**	**SE**	**95%** **CI**		**t**	***p***	**b**	**SE**	**95%** **CI**		**t**	***p***
Sex (Male)	0.35	0.07	0.21	0.49	4.88	0.000	0.54	0.13	0.28	0.79	4.13	0.000
Age	−0.23	0.07	−0.37	−0.09	−3.19	0.001	−0.10	0.13	−0.35	0.14	−0.81	0.415
Household Size	−0.01	0.03	−0.06	0.04	−0.44	0.662	0.00	0.04	−0.08	0.07	−0.06	0.953
Married household	−0.25	0.10	−0.45	−0.05	−2.49	0.013	−0.24	0.15	−0.52	0.05	−1.63	0.103
Subjective Family SES	−1.18	0.30	−1.77	−0.58	−3.86	0.000	0.16	0.30	−0.43	0.76	0.54	0.586
Constant	8.86	0.75	7.40	10.33	11.88	0.000	6.78	1.24	4.35	9.21	5.46	0.000

b = Unstandardized Regression Coefficient, SE = Standard Error, CI = Confidence Interval, SES = Socioeconomic Status.
